# *Mycoplasma pneumoniae* Seroprevalence and Total IgE Levels in Patients with Juvenile Idiopathic Arthritis

**DOI:** 10.1155/2021/6596596

**Published:** 2021-10-06

**Authors:** Dimitri Poddighe, Diyora Abdukhakimova, Kuanysh Dossybayeva, Zaure Mukusheva, Maykesh Assylbekova, Marzhan Rakhimzhanova, Aigul Ibrayeva, Gaukhar Mukash, Yernas Tuleutayev

**Affiliations:** ^1^Department of Medicine, Nazarbayev University School of Medicine, Nur-Sultan, Kazakhstan; ^2^Clinical Academic Department of Pediatrics, National Research Center for Maternal and Child Health, Nur-Sultan, Kazakhstan

## Abstract

**Background:**

*Mycoplasma pneumoniae* (*M. pneumoniae*) is implicated in several immune-mediated extrapulmonary manifestations, including reactive arthritis. Recently, increased total serum IgE were reported in children developing *M. pneumoniae*-related extrapulmonary diseases (MpEPDs). Here, we aimed at analyzing these aspects in children affected with rheumatic disorders and, in detail, Juvenile Idiopathic Arthritis (JIA).

**Methods:**

*M. pneumoniae* serology (IgG and IgM) and total serum IgE were concomitantly analyzed in 139 pediatric patients diagnosed with: JIA (Group 1, *n* = 85), or any rheumatic disease other than JIA (Group 2, *n* = 27), or non-inflammatory endocrinological disorders (Group 3, *n* = 27).

**Results:**

Overall, 19.4% *M. pneumoniae* seroprevalence was observed in this hospitalized pediatric population, without signicant differences among the three groups. No significant differences in total serum IgE levels were noted among these groups; however, a second analysis excluding children with very high (and clearly abnormal) IgE levels suggested that JIA patients and, in detail, those with oligopolyarticular forms may have higher serum IgE concentrations. This relative difference among groups in serum IgE level seems to be more pronounced in *M. pneumoniae* seropositive children.

**Conclusions:**

*M. pneumoniae* infection should be actively sought in children developing immune-mediated diseases, including patients affected with JIA and, especially, in oligopolyarticular forms. There is some evidence that total serum IgE levels may tend to be increased in patients with oligopolyarticular JIA subtype and especially in those resulting as *M. pneumoniae* seropositive. However, further and focused research is needed to confirm these preliminary results and to clarify the relation between *M. pneumoniae* infection, atopic status, and immune-mediated arthritis.

## 1. Introduction

*Mycoplasma pneumoniae* (*M. pneumoniae*) belongs to the class of bacteria named as *Mollicutes*, which are characterized by the absence of a cell wall around their cell membrane. In terms of cellular size and genome length, these microorganisms are the smallest free-living and self-replicating bacteria [1]. Among the approximately 100 species included into this bacterial class, *M. pneumoniae* plays a leading role from a medical point of view. Indeed, *M. pneumoniae* causes a wide range of respiratory diseases in humans, including upper respiratory tract infections, tracheitis/bronchitis, and pneumonia [2].

Moreover, *M. pneumoniae* infection can also manifest with several extra-respiratory manifestations. Indeed, respiratory manifestations can be mild and, thus, may be completely overlooked. Therefore, it is not unusual (especially in children) that such an infection is diagnosed because of some *M. pneumoniae*-related extra-pulmonary diseases (MpEPDs). MpEPDs can affect several organs and systems (e.g., joints, muscles, skin, mucosae, blood cells, and heart) and are supposed to mainly rely on immune-mediated mechanisms, which are still largely unclear and are likely to be multiple, considering the number of different clinical manifestations that have been described so far as linked to *M. pneumoniae* infection [3, 4].

The musculoskeletal system, including joints, is the most frequent target of MpEPDs, along with the skin and mucosae [3, 4]. Moreover, several reports implicated *M. pneumoniae* in patients with rheumatoid arthritis, and in some cases, its genetic material was detected in the synovial fluid [5–7]. In children, autoimmune chronic arthritis is named as Juvenile Idiopathic Arthritis (JIA), which includes all types of chronic arthritis with no apparent cause, in patients younger than 16 years at onset. According to the International League of Associations for Rheumatology (ILAR), five main subtypes can be defined inside the JIA classification: systemic (sJIA); oligoarticular (oJIA), which may be persistent or extended; polyarticular (pJIA), which is usually rheumatoid factor (RF) negative and, much less frequently, positive; psoriatic (PsJIA); and enthesitis-related (ERA). Additionally, JIA may be categorized as undifferentiated, if arthritis does not fulfill the diagnostic criteria for any of the aforementioned subtypes [8]. Although JIA is idiopathic by definition, its pathogenesis in genetically susceptible individuals may be triggered by the exposure to some unclear environmental factors, including infectious agents. Some authors suggested the potential role of bacteria in JIA pathogenesis, and *M. pneumoniae* has been considered, too [9, 10].

Recently, we reported an increased frequency of atopy (defined as increased levels/production of total serum IgE) in children diagnosed with MpEPDs, which may lead to speculate that IgE or, more likely, the immunological environment behind this phenotype, might be partially implicated in the immune pathogenesis of MpEPDs, including articular and musculoskeletal manifestations [11, 12]. In this regard, the role of IgE (as well as the potential implication of *M. pneumoniae*) in JIA has been poorly investigated so far. Increased levels of serum IgE were previously reported in patients affected with rheumatoid arthritis, where some authors also suggested the presence of IgE autoantibodies (e.g., ANA and RF) [13, 14]. However, no studies regarding this aspect in rheumatoid arthritis and/or JIA have been recently published, but the presence of self-reactive IgE has been reported in a growing number of autoimmune disorders, where those seem to be present in a significant portion of patients [15, 16].

In this preliminary research, we aimed at providing some initial and integrated observations on both *M. pneumoniae* infection and serum IgE in patients with JIA, compared to children with other rheumatic and non-rheumatic disorders.

## 2. Patients and Methods

This cross-sectional study investigated *Mycoplasma pneumoniae* serology and total serum IgE in 139 pediatric patients admitted to the National Research Center for Maternal and Child Health (NRCMCH) of the University Medical Center (UMC), affiliated with the Nazarbayev University School of Medicine (NUSOM) in Nur-Sultan (Kazakhstan).

This study and its procedures were approved after full board review by both the Institutional Research Ethical Committee of the Nazarbayev University (application no. 186/14102019, approved on February 17^th^, 2020) and the Institutional Review Board of UMC (application no. 005-2020, approved on May 25^th^, 2020). This study included all pediatric patients whose guardians gave the consent to donate a small aliquot of blood for research purposes, in the period comprised between July and November 2020. In this period, 139 patients were enrolled as belonging to one of the following study groups: patients diagnosed with JIA (Group 1), children diagnosed with any rheumatic disease other than JIA (Group 2), and children affected with non-inflammatory disorders admitted to the ward of general pediatrics (Group 3). The inclusion of these patients (Group 3) aimed to provide a “control” group including children affected with non-inflammatory disorders, to be compared with inflammatory rheumatic (both JIA and non-JIA) diseases. Our ethical approval did not allow us to include completely healthy children in the present study, because only patients undergoing venipuncture for own medical reasons were eligible to be recruited.

The available secondary data about demographic, clinical, and laboratory characteristics were retrieved from patients' clinical records. Unfortunately, the clinical files were not complete as regards concomitant and/or previous allergic disorders. In general, the data were collected in Excel file format, and the quantitative variables are expressed as mean values (±standard deviation, SD) for the descriptive statistics. The IgM and IgG specific to *M. pneumoniae* were assessed by enzyme-immunoassay (*Mycoplasma pneumoniae*-IgM-EIA-BEST kit and *Mycoplasma pneumoniae*-IgG-EIA-BEST, Vector Best, Novosibirsk, Russia); the results were expressed as optical density (OD) coefficient, and a value > 0.9 was considered as a positive titer, as indicated by our clinical laboratory. The serum concentration of total IgE was measured by immune-chemiluminescence (Elecsys Total IgE II immunoassay, Roche). IgE levels were expressed as IU/ml, and the age-related reference values were provided by our clinical laboratory, as follows: <30 IU/ml (0-3 years), <60 IU/ml (3-7 years), <90 IU/ml (7-10 years), <100 IU/ml (10-13 years), 150 IU/ml (13-15 years), and <100 IU/ml (16-17 years).

The statistical analysis of the differences in quantitative variables between two groups was made by using the GraphPad Prism® software, and in detail, unpaired one-tail *t*-test with Welch's correction was used (*p* value < 0.05 was considered as statistically significant), assuming unequal variance and considering the unequal sample sizes. The statistical analysis of event frequencies between two groups was assessed by using the GraphPad Prism® software, and in detail, the *χ*^2^-test was used (*p* value < 0.05 was considered as statistically significant).

## 3. Results

### 3.1. Demographic and Clinical Characteristics of the Study Population and Groups

A total of 139 pediatric patients were enrolled in this study: among them, 85 were diagnosed with JIA (JIA patients: Group 1), 27 patients were affected with rheumatic disorders other than JIA (non-JIA patients: Group 2), and 27 children had endocrine disorders (non-rheumatic patients, Group 3: diabetes mellitus type 1, *n* = 21; growth hormone deficiency, *n* = 3; thyrotoxicosis, *n* = 2; and Turner syndrome, *n* = 1). Among the JIA patients, the following subtypes were represented: oJIA (*n* = 54), pJIA (*n* = 13), RF+pJIA (*n* = 2), sJIA (*n* = 8), ERA (*n* = 4), and PsJIA (*n* = 4). As regards the non-JIA rheumatic children, the following rheumatic conditions were diagnosed: localized scleroderma (*n* = 12), pediatric systemic lupus erythematosus (*n* = 7), juvenile dermatomyositis (*n* = 3), Behcet's disease (*n* = 3), acute rheumatic fever (*n* = 1), and systemic sclerosis (*n* = 1). The demographic characteristics of these three study groups are summarized in Table 1

### 3.2. Assessment of *M. pneumoniae* Serology and IgE Levels in Rheumatic Children

All these 139 pediatric patients were serologically screened to assess recent and/or past *M. pneumoniae* infections: 27 patients (19.4%) tested positive for IgM and/or IgG (OD coefficient > 0.9) specific to *M. pneumoniae*. Among those *M. pneumoniae* serologically positive children, 18 patients belonged to Group 1 (JIA patients), whereas the remaining 9 positive children were distributed between Group 2 (*n* = 4) and Group 3 (*n* = 5). Therefore, the percentages of *M. pneumoniae* seropositivity in the three groups were 21.2%, 14.8%, and 18.5%, respectively; however, these differences were not statistically significant (*p* > 0.05).

In addition to the overall results of the *M. pneumoniae* serology, Table 2 also shows the values of total IgE in serum. Serum IgE values were expressed as both absolute (as measured; total IgE) and relative (expressed as ratio between the actual absolute value and the upper age-related normal limit; IgE ratio) values. No statistically significant differences were observed among these three groups (*p* > 0.05).

However, we tried to reanalyze these data by excluding the “outliers” characterized by extremely elevated serum IgE level (that we defined as a value 6 times higher than the upper age-related normal limit). The rationale of excluding these patients was to reduce the interference of patients who may be affected with concomitant and/or preexisting allergic disorders, which may not be diagnosed due to the limitations of the allergy work-up in local and/or national hospital settings. After this correction, Group 1 showed an absolute value of IgE (mean ± standard error of the mean, M ± SEM) of 103.90 ± 13.92 IU/ml, which resulted to be higher than Group 2 (61.15 ± 14.30 IU/ml; *p* = 0.0178) and Group 3 (70.34 ± 21.99 IU/ml; *p* > 0.05). These differences resulted to be both statistically significant when the IgE ratio (which could reduce the effect of the age on the expected normal level of total IgE) instead of the absolute serum IgE level, was considered (M ± SEM; Group 1 vs. Group 2: 1.139 ± 0.158 vs. 0.648 ± 0.150, respectively, *p* = 0.0137; Group 1 vs. Group 3: 1.139 ± 0.158 vs. 0.661 ± 0.180, respectively, *p* = 0.0253).

### 3.3. Prevalence of Rheumatic Disorders and IgE Levels in Children Seropositive for *M. pneumoniae*

Table 3 provides a more detailed overview of those 27 *M. pneumoniae* serologically positive patients. Among the 18 *M. pneumoniae* seropositive children affected with JIA, the subtype distribution was as follows: oJIA, *n* = 13; pJIA, *n* = 3; RF+pJIA, *n* = 1; and sJIA, *n* = 1. As regards the remaining 9 MP-positive patients, 4 and 5 children belonged to the non-JIA rheumatic Group 2 (pSLE, *n* = 2; BD, *n* = 1; and localized scleroderma, *n* = 1) and the non-rheumatic Group 3 (T1DM, *n* = 4; GHD, *n* = 1).

No statistically significant differences in serum IgE levels (expressed as total concentration or IgE ratio) were present between *M. pneumoniae* positive and negative patients in each of the three groups (I, II, and III). Interestingly, the serum IgE levels of Group 1 *M. pneumoniae* seropositive patients were significantly higher than those of the non-Group 1 *M. pneumoniae* positive patients (M ± SEM, Group 1 vs. Groups 2 + 3; total IgE absolute level: 149.8 ± 32.50 vs. 44.39 ± 13.15 IU/ml, respectively, *p* = 0.0034; IgE ratio: 1.455 ± 0.336 vs. 0.532 ± 0.196, respectively, *p* = 0.0379), even considering Group 2 (M ± SEM, Group 1 vs. Group 2; total IgE absolute level: 149.8 ± 32.50 vs. 37.24 ± 24.83 IU/ml, respectively, *p* = 0.0078; IgE ratio: 1.455 ± 0.336 vs. 0.557 ± 0.431, respectively, *p* = ns) and Group 3 (M ± SEM, Group 1 vs. Group 3; total IgE absolute level: 149.8 ± 32.50 vs. 50.11 ± 15.30 IU/ml, respectively, *p* = 0.0058; IgE ratio: 1.455 ± 0.336 vs. 0.511 ± 0.163, respectively, *p* = 0.01) separately. Interestingly, this association between JIA and the higher total serum IgE levels seems to persist even when all patients (both seropositive and seronegative) were considered. As shown in Table 4, some association between higher corrected total serum IgE levels and oligopolyarticular forms of JIA is still present, regardless of the *M. pneumoniae* serology status.

## 4. Discussion

In this cross-sectional study, one objective was to assess the seroprevalence to *M. pneumoniae* infection in the pediatric population from Kazakhstan. Indeed, there are no available studies exploring the implication of *M. pneumoniae* in respiratory diseases from Central Asia, although this pathogen has been increasingly recognized as implicated in pediatric community-acquired pneumonia in other Asian regions [17, 18]. Overall, 19.4% of the 139 children included in this study resulted to be serologically positive for *M. pneumoniae*. A study from Taiwan showed that 19.84% of healthy Taiwanese adolescents were seropositive for *M. pneumoniae* [19]. In China, the prevalence of *M. pneumoniae* infections was reported as high as 18.8%, among adult and adolescent patients with respiratory tract infection [20]. Ranjbar and Halaji recently suggested that the seroprevalence of *M. pneumoniae* in Iran and, more in general, in several Asian countries, is comparable to other parts of the world, based on their meta-analysis [21]. Our findings suggest that *M. pneumoniae* is as prevalent in Kazakhstani children as in other Asian pediatric populations. Therefore, this infection should be assessed in children with atypical extra-pulmonary immune-mediated manifestations and may be also part of the infectious work-up preceding the diagnosis of JIA, which should be differentiated from reactive arthritis.

Importantly, our study participants were not selected according to the presence of any respiratory diseases: as explained, the study groups included hospitalized patients with JIA, rheumatic disorders other than JIA, and endocrinological and non-inflammatory conditions. In these three groups, the seroprevalence rates for *M. pneumoniae* infection was not statistically different.

As already mentioned, the diagnosis of *M. pneumoniae* infection may be overlooked in a remarkable number of patients, since the respiratory symptoms can be mild, and the infection is not necessarily associated with pneumonia [2, 4]. Indeed, *M. pneumoniae* infection can be detected during the work-up for extra-respiratory conditions, namely MpEPDs [3, 4]. Several reports showed that reactive arthritis can be associated with *M. pneumoniae* infection, especially in children [22–24]. A study by Azumagawa et al. showed that arthritis in the absence of pneumonia may not be an unusual occurrence in pediatric patients infected by *M. pneumoniae* [25]. Previously, our group also described children with arthritis who were finally diagnosed as having reactive diseases after *M. pneumoniae* infection, among patients affected with a variety of MpEPDs (including vasculitis-related urticarial rashes, myositis, nephritis, and myocarditis/pericarditis). Moreover, we also observed increased serum levels of total IgE in these children developing MpEPDs, which was not related to any concomitant respiratory and/or allergic diseases [11, 12]. Therefore, we decided to concomitantly analyze these aspects (*M. pneumoniae* seroprevalence and total serum IgE) in children affected with JIA (Group 1), compared to pediatric patients with different rheumatic disorders (Group 2) and without any rheumatic-inflammatory diseases (Group 3). In this regard, we did not find any statistically significant differences among these three groups by using the raw data, which may be due to both the limited sample sizes and the potential interference of coexisting allergic diseases, which could not be excluded completely, as previously explained. Therefore, we tried to bypass or reduce this potential problem by reanalyzing our data after exclusion of patients with very high total IgE levels (>6 times the upper age-related limit), which would strongly suggest some underlying allergic diseases, parasite infections, or other acquired hyper-IgE disorders that may have been overlooked. This way, we noticed mild statistically significant differences in corrected total IgE levels between JIA patients and the other two groups, especially considering the IgE ratio, which also lessens the impact of the age factor on the total IgE levels in serum.

Conversely, no remarkable differences were observed in terms of *M. pneumoniae* seroprevalence among these three groups. However, *M. pneumoniae* seropositive Group 1 patients showed significantly higher total IgE levels than the seropositive children belonging to Group 2 and Group 3. This finding could suggest that the effect of previous/recent infection by *M. pneumoniae* on serum IgE levels may differ according to the clinical background. However, it is not possible to conclude about any cause-effect and/or temporal relation between *M. pneumoniae* infection and IgE levels in JIA, due to the cross-sectional design of this study. Interestingly, some association between higher total serum IgE levels (after exclusion of "outliers") and oligopolyarticular forms of JIA is still present regardless of the *M. pneumoniae* serology status.

This aspect of higher IgE levels (atopy) in children developing MpEPDs was noticed by other authors. Recently, Wang et al. reported a higher rate of atopy in children hospitalized for *M. pneumoniae* pneumonia, who concomitantly or eventually developed extra-pulmonary manifestations [26]. However, *M. pneumoniae* by itself may increase IgE production in some individuals [27]; on the contrary, a preexisting atopic status may work as a risk factor to develop complicated forms of *M. pneumoniae* infections, including MpEPDs [28].

Therefore, the understanding of the relationship between *M. pneumoniae* infection/atopy (and their interplay) and the development of MpEPDs (or even some autoimmune diseases, like JIA) absolutely requires additional and focused research. Indeed, this study is affected by several and important limitations, including the absence of a completely healthy pediatric population to compare our rheumatic patients. However, the Group 3 can represent a good term of comparison versus rheumatic patients (JIA and non-JIA). Moreover, the samples size is relatively small, and the additional analysis regarding the total IgE levels and IgE ratio was performed by excluding patients with very high IgE levels, according to a clinical definition rather than a statistical calculation of outliers. Therefore, clinical studies based on an appropriate selection and standardization of the study population (taking into account the preliminary observations emerging from the present study) and with a longitudinal follow-up of participants (indeed, our study was limited by the cross-sectional design, and the antibody titers are variable during the course of infection) are needed. Finally, the use of both indirect (serological) and direct (ideally, PCR-based) microbiological analyses specific to *M. pneumoniae* would provide additional scientific value to future studies.

## 5. Conclusion

We showed that the seroprevalence of *M. pneumoniae* infection in Kazakhstan (Central Asia) is not negligible (19.4%, in our case series of hospitalized children) and, therefore, should be actively sought in children developing immune-mediated diseases. This is especially true for patients affected with JIA and, especially, in oligopolyarticular forms. Moreover, there is some initial and preliminary evidence that total serum IgE levels may tend to be increased in patients with oligopolyarticular JIA subtypes, and this aspect seems to be more pronounced in *M. pneumoniae* seropositive oJIA patients. However, due to the several limitations of this research, further and focused studies are absolutely needed to confirm these preliminary results and appropriately clarify the relation between *M. pneumoniae* infection, atopic status, and immune-mediated arthritis.

## Figures and Tables

**Table 1:** Clinical and demographic characteristics of the study participants.

	Group 1 (rheumatic JIA) *N* = 85	Group 2 (rheumatic non-JIA) *N* = 27	Group 3 (non-rheumatic) *N* = 27	Total (all patients) *N* = 139
*Gender*				
Male	27	8	13	48
Female	58	19	14	91
*Age*				
0-5 years	7	1	5	13
6-10 years	34	8	4	46
>10 years	46	18	18	82
Mean (±SD)	10.7 ± 4.1	11.4 ± 3.5	11.0 ± 4.6	10.9 ± 4.1
*Diagnosis*				
oJIA	54	—	—	54
pJIA	13	—	—	13
RF+JIA	2	—	—	2
sJIA	8	—	—	8
PsJIA	4	—	—	4
ERA	4	—	—	4
Loc. SCD	—	12	—	12
Syst. SCL	—	1	—	1
pSLE	—	7	—	7
JDM	—	3	—	3
BD	—	3	—	3
ARF	—	1	—	1
T1DM	—	—	21	21
GHD	—	—	3	3
Thyrotoxicosis	—	—	2	2
Turner syndrome	—	—	1	1
*Resp. comorbidity*				
RRI	4	2	1	7
Asthma	2	1	1	4
Previous pneumonia	5	1	—	6
*Atopic comorbidity*				1
AR	—	—	1	1
AD	1	—	—	1
Drug allergy	1	—	—	1
Food allergy	1	—	—	1

Abbreviations: JIA: juvenile idiopathic arthritis; oJIA: oligoarticular JIA; pJIA: polyarticular JIA; RF: rheumatoid factor; sJIA: systemic JIA; PsJIA: psoriatic JIA; ERA: enthesis-related arthritis; SCD: scleroderma; Syst. SCL: systemic sclerosis; pSLE: pediatric systemic lupus erythematosus; BD: Behcet's disease; ARF: acute rheumatic fever; T1DM: type 1 diabetes mellitus; GHD: growth hormone deficiency; RRI: recurrent respiratory infections; AR: allergic rhinitis; AD: atopic dermatitis.

**Table 2:** *Mycoplasma pneumoniae* serology and total serum IgE levels in the study population and groups.

	Group 1 (rheumatic JIA) *N* = 85	Group 2 (rheumatic non-JIA) *N* = 27	Group 3 (non-rheumatic) *N* = 27	Total (all patients) *N* = 139
*MP serology*				
-IgG titer (OD coeff.)	0.41 ± 0.92	0.31 ± 0.68	0.30 ± 0.43	0.37 ± 0.80
-IgM titer (OD coeff.)	0.59 ± 0.56	0.59 ± 0.70	0.50 ± 0.62	0.57 ± 0.60
Positive MP serology	18	4	5	27
-IgG titer (OD coeff.)	1.42 ± 1.66	1.23 ± 1.38	0.80 ± 0.75	1.27 ± 1.46
-IgM titer (OD coeff)	1.26 ± 0.84	1.45 ± 1.54	1.05 ± 1.46	1.30 ± 0.99
Negative MP serology	67	23	22	112
*Serum IgE level*				
Total IgE (IU/ml)	244.2 ± 549.0	171.4 ± 426.4	384.1 ± 1358.6	257.2 ± 755.1
IgE ratio	2.86 ± 6.93	1.79 ± 4.36	2.83 ± 9.07	2.65 ± 6.96
Total IgE^∗^ (IU/ml)	103.9 ± 122.9	61.2 ± 71.5	70.3 ± 107.7	86.9 ± 110.2
IgE ratio^∗^	1.14 ± 1.40	0.65 ± 0.75	0.66 ± 0.88	0.91 ± 1.14

Abbreviations: MP: *Mycoplasma pneumoniae*; OD coeff.: optical density coefficient; JIA: juvenile idiopathic arthritis. ^∗^Total IgE and IgE ratio, after exclusion of the “outliers,” as explained in the manuscript.

**Table 3:** Main demographic, clinical, and laboratory parameters in *M. pneumoniae* serologically positive children.

Group	Pt.	Gender	Age	Diagnosis	MP-IgM	MP-IgG	Total IgE	IgE limit	Notes	IgE ratio
I	1	F	16.0	oJIA	2.26	1.79	106.40	*100*	—	1.064
	2	F	9.2	oJIA	0.71	4.44	212.20	*100*	P	2.358
	3	F	11.8	oJIA	0.71	3.36	368.60	*100*	DA	3.686
	4	F	13.1	oJIA	0.45	4.67	255.10	*150*	—	1.701
	5	M	12.8	oJIA	1.01	0.01	5.99	*150*	RRI	0.060
	6	M	2.7	oJIA	1.00	0.24	14.85	*30*	—	0.495
	7	F	10.6	oJIA	1.96	0.36	83.00	*100*	—	0.830
	8	F	7.6	oJIA	0.99	0.30	21.72	*90*	RRI	0.241
	9	M	6.9	oJIA	1.05	0.23	1.07	*60*	—	0.018
	10	M	14.9	oJIA	2.94	0.62	21.71	*150*	—	0.145
	11	M	9.2	oJIA	0.92	0.32	379.00	*90*	—	4.211
	12	M	15.7	oJIA	1.10	0.02	205.10	*100*	P	2.051
	13	F	13.3	oJIA	0.94	0.12	237.30	*150*	—	1.582
	14	F	7.8	pJIA	0.74	3.59	5.53	*90*	—	0.061
	15	F	14.5	pJIA	0.35	2.85	16.38	*150*	A	0.109
	16	M	12.6	pJIA	1.10	0.30	274.50	*100*	—	2.745
	17	M	14.3	RF+pJIA	3.44	0.57	132.00	*150*	—	0.880
	18	F	7.7	sJIA	0.92	0.43	355.90	*90*	P	3.954

II	19	F	5.1	Loc. SCD	1.06	0.02	110.70	*60*	—	1.845
	20	F	15.4	pSLE	0.12	2.01	22.31	*100*	—	0.223
	21	F	12.2	pSLE	0.97	0.12	13.78	*150*	—	0.138
	22	F	16.4	BD	3.86	2.78	2.16	*100*	—	0.022

III	23	M	2.0	T1DM	0.66	1.01	8.17	*30*	—	0.272
	24	M	17.8	T1DM	0.61	1.01	79.26	*100*	—	0.793
	25	F	14.6	T1DM	1.72	0.09	36.17	*150*	—	0.241
	26	F	14.2	T1DM	3.16	0.04	35.92	*150*	—	0.239
	27	M	9.1	GHD	0.37	1.84	91.05	*100*	—	1.012

Abbreviations: MP: *Mycoplasma pneumoniae*; Pt.: patient; JIA: juvenile idiopathic arthritis; P: personal history of pneumonia; oJIA: oligoarticular JIA; pJIA: polyarticular JIA; RF: rheumatoid factor; sJIA: systemic JIA; SCD: scleroderma; pSLE: pediatric systemic lupus erythematosus; BD: Behcet's disease; T1DM: type 1 diabetes mellitus; GHD: growth hormone deficiency; RRI: recurrent respiratory infections; A: unspecified diagnosis of allergy; AD: atopic dermatitis.

**Table 4:** *M. pneumoniae* serology titers and total serum IgE level in oligopolyarticular JIA patients compared to non-JIA study groups.

	Total IgE^∗^	Total IgE ratio^∗^	MP IgG titer	MP IgM titer
oJIA+pJIA	109.5 ± 135.6	1.12 ± 1.33	0.48 ± 1.01	0.64 ± 0.60
Group 2	61.2 ± 71.5	0.65 ± 0.75	0.31 ± 0.68	0.59 ± 0.70
*p*	*0.0158*	*0.0197*	*0.1684*	*0.3961*
Group 3	70.3 ± 107.7	0.66 ± 0.88	0.30 ± 0.43	0.50 ± 0.62
*p*	0.0818	*0.0335*	*0.1289*	*0.1909*

^∗^Total IgE and IgE ratio, after exclusion of the “outliers,” as explained in the manuscript.

## Data Availability

Due to the nature of this research, participants of this study did not agree for their data to be shared publicly.
